# Confinement of Oligomeric Vinyl Sulfonic Acid Within Crosslinked Porous Polybenzimidazole for Intermediate-Temperature Proton Exchange Membranes

**DOI:** 10.3390/polym18111298

**Published:** 2026-05-25

**Authors:** Hongbin Na, Sung-Kon Kim

**Affiliations:** Department of Chemical and Biochemical Engineering, Dongguk University, Seoul 04620, Republic of Korea; 2026121074@dgu.ac.kr

**Keywords:** proton exchange membrane, vinyl sulfonic acid, polybenzimidazole, intermediate temperature, proton conductivity

## Abstract

This study reports the intermediate-temperature proton exchange membrane (IT-PEM) based on an oligomeric vinyl sulfonic acid (OVS)-infiltrated crosslinked porous polybenzimidazole (cp-PBI) framework. The cp-PBI membrane, fabricated via ZIF-8-templated porosity and covalent crosslinking, provides a mechanically robust and chemically stable host matrix that enables high uptake and uniform distribution of OVS throughout the membrane bulk. In situ oligomerization of vinyl sulfonic acid yields a wax-like OVS ionomer with high proton density and reduced mobility, effectively suppressing ionomer leaching while maintaining efficient proton transport under anhydrous conditions. The resulting membrane exhibits high proton conductivity of 8.4 × 10^−3^ S cm^−1^ at room temperature and 2.6 × 10^−2^ S cm^−1^ at 110 °C without any external humidification. Compared to dense PBI and conventional phosphoric acid (PA)-doped systems, the composite membrane demonstrates significantly enhanced ionomer retention, with only 2.3 wt% loss under compressive conditions and improved stability under humid environments. These results highlight the synergistic effect of a porous crosslinked host and viscous oligomeric ionomer, providing a promising strategy for designing stable, high-performance IT-PEMs.

## 1. Introduction

Proton exchange membranes (PEMs) are key components in electrochemical energy systems, functioning as solid electrolytes that selectively conduct protons while blocking electrons and reactant gases [1,2,3]. They enable efficient ion transport and maintain separation between fuel and oxidant, thereby governing the overall performance and durability of the system. While PEMs have traditionally been developed for either low-temperature or high-temperature operation, increasing attention is being directed toward membranes that can operate effectively in the intermediate-temperature regime. Conventional low-temperature PEMs (LT-PEMs), typically operating below 80 °C, rely on fully humidified conditions to sustain proton conductivity in perfluorosulfonic acid membranes such as Nafion [4,5,6]. However, their performance is fundamentally limited by sluggish oxygen reduction kinetics, severe water management issues (flooding and drying), and poor tolerance to carbon monoxide impurities [7,8,9]. On the other hand, high-temperature PEMs (HT-PEMs), operating in the range of 120–200 °C, offer improved reaction kinetics, enhanced CO tolerance, and simplified water management due to reduced dependence on external humidification [10,11,12]. Despite these advantages, HT-PEMs typically rely on alternative electrolyte systems such as phosphoric acid (PA)-doped membranes, which introduce critical challenges including acid leaching, chemical degradation, and limited long-term durability [13,14].

In this context, intermediate-temperature PEMs (IT-PEMs), operating in the range of 80–120 °C, have emerged as a highly attractive alternative, as they bridge the gap between LT- and HT-PEMs while mitigating the limitations of both systems. At these temperatures, electrode kinetics—particularly for the oxygen reduction reaction (ORR)—are significantly enhanced compared to LT-PEMs, leading to reduced activation overpotential and improved overall efficiency [15,16]. Simultaneously, the elevated temperature improves CO tolerance by promoting CO desorption from catalyst surfaces, enabling the use of reformate hydrogen without extensive purification. From a transport and operational standpoint, the intermediate-temperature regime provides distinct advantages. It alleviates the critical water management challenges of LT-PEMs by promoting water evaporation, thereby reducing cathode flooding, while still retaining sufficient hydration for proton conduction [17,18]. This results in stable operation over a broader humidity range. In addition, IT-PEMs enable more efficient utilization of waste heat and simplified thermal management compared to low-temperature systems, while avoiding the severe material degradation, acid leaching, and mechanical instability commonly encountered in HT-PEMs (>120 °C) [19,20].

Importantly, the intermediate-temperature regime imposes unique and stringent requirements on membrane materials. The membrane must simultaneously sustain sufficiently high proton conductivity under low-humidity or anhydrous conditions and maintain long-term electrolyte retention and structural integrity, which is non-trivial for conventional Nafion-type or PA-doped systems. Conventional perfluorosulfonic acid membranes suffer from dehydration and a sharp decline in proton conductivity above ~80 °C under low-humidity conditions, whereas PA-doped, while widely used for high-temperature PEMFCs, typically exhibit strong temperature-dependent acid leaching and mechanical softening, leading to rapid loss of conductivity and durability in the intermediate-temperature window [21,22,23]. As a result, the development of IT-PEMs has driven the exploration of alternative electrolyte systems, including composite membranes, acid-doped polymers, and ionically functionalized materials, designed to simultaneously achieve high proton conductivity, reduced humidity dependence, and robust interfacial stability [24,25,26]. Recent studies on sulfonic-acid-functionalized PBI systems have shown that introducing fixed acid sites can improve anhydrous proton transport and mitigate free acid loss; however, achieving a high density of acid functionalities without sacrificing mechanical robustness and ion retention remains challenging [27,28]. Despite these efforts, only a limited number of PEM materials have demonstrated reliable and high proton conductivity in the intermediate-temperature range under practical (low-humidity or anhydrous) conditions. Therefore, the development of advanced PEMs specifically tailored for intermediate-temperature operation remains a critical challenge and an essential step toward realizing high-performance, durable, and system-efficient fuel cell technologies [29,30].

In this study, oligomeric vinyl sulfonic acid (OVS)-infiltrated crosslinked porous polybenzimidazole (cp-PBI) framework was prepared for the use in IT-PEMs. Combining a crosslinked porous PBI framework with a confined oligomeric sulfonic acid phase offers a promising route to decouple mechanical support from proton conduction. The cp-PBI membrane, prepared via a zeolite imidazolate framework-8 (ZIF-8)-templated porous structure and covalent crosslinking, serves as a robust host matrix enabling high uptake and uniform distribution of OVS throughout the membrane bulk. By controlling the in situ oligomerization of vinyl sulfonic acid, a wax-like ionomer with high proton density and suppressed mobility was obtained, facilitating efficient proton transport under anhydrous conditions while minimizing ionomer leaching. The resulting membrane exhibits high proton conductivity over a wide temperature range, including the intermediate-temperature regime (80–120 °C), without the need for external humidification or PA doping. Furthermore, the membrane demonstrates significantly improved ionomer retention and stability under both compressive and humid conditions compared to conventional PA-doped polybenzimidazole systems. These findings highlight a promising strategy for designing stable and highly conductive PEMs specifically tailored for intermediate-temperature operation.

## 2. Materials and Methods

### 2.1. Preparation of Composite Membranes

The synthesis, film fabrication, and PA doping of poly [2,2′-(*m*-phenylene)-5,5′-bibenzimidazole] (PBI), as well as the preparation of crosslinked porous PBI (cp-PBI), were conducted following our previously reported methods [31]. Briefly, for cp-PBI, a 10 wt% PBI solution in DMAc was mixed with methylene diphenyl diisocyanate (MDI) and ZIF-8 (10 wt% relative to PBI + MDI) in the presence of a small amount of triethylamine, cast onto a quartz substrate, and thermally treated to obtain self-supporting films. The resulting films were thoroughly washed with water, dried under vacuum, and finally doped by immersion in 85 wt% PA at 80 °C for 4 h. The films were taken out of the solution and washed with deionized water several time to remove PA, if any, followed by drying at room temperature for 24 h in a vacuum oven. The dried films (i.e., cp-PBI) with a thickness of 40 μm were subsequently immersed in an *N*,*N*-dimethylacetamide (DMAc, 99.5%, Samchun, Republic of Korea) solution containing 1 M vinylsulfonic acid (VSA, >97%, TCI) and 0.1 wt% ammonium persulfate (APS, ≥98%, Sigma-Aldrich, USA) as the radical initiator, and reacted at 60 °C for 24 h. The membranes were then taken out of the reaction solution and gently blotted with filter paper to remove surface solvent. The resulting films were dried at 25 °C for 12 h under vacuum, resulting in OVS-infiltrated cp-PBI composite membranes (termed OVS-cp-PBI, ~50 μm of thickness).

### 2.2. Characterization

Field-emission scanning electron micrographs (FE-SEMs) were obtained using a Carl Zeiss SUPRA 40VP microscope, which was coupled to an energy-dispersive X-ray (EDX) detector to probe the elemental composition. Matrix-assisted laser desorption/ionization time-of-flight mass spectra (MALDI-TOF MS) were acquired on a Voyager-DE STR Biospectrometry Workstation (Applied Biosystems Inc. USA) operated in positive reflection mode with dithranol as the matrix. The instrument employed a nitrogen laser (λ = 337 nm, pulse width ≈ 3 ns). Thermal stability was assessed by thermogravimetric analysis (TGA, SCINCO N-1000, Republic of Korea) under nitrogen flow (10 °C min^−1^). Impedance measurements were performed with a single-channel potentiostat/impedance analyzer (SP-200, BioLogic, France) operated in potentiostatic mode, applying a 10 mV AC perturbation over the 7 MHz–10 mHz frequency range under anhydrous conditions. To establish a dry environment, the measurement cell was purged with nitrogen gas before and during the test. The membrane was sandwiched between the reference and sensing electrodes, and the impedance response was recorded in the through-plane direction. The intercept of the Nyquist plot on the real (*Z′*) axis was taken as the ohmic resistance (*R*) of the membrane. Proton conductivity was determined using through-plane configuration and calculated according to the following relation:

(1) $$\sigma =\frac{T}{RWL}$$

where *σ* represents proton conductivity, *L* is the distance between the reference and sensing electrodes, and *W* and *T* denote the width and thickness of the electrolyte membrane, respectively. The activation energy (*E*_a_) was evaluated from the temperature-dependent ionic conductivity, derived from the bulk resistance, according to the Arrhenius equation:

(2) $$\sigma_{T}=Aexp(\frac{{-E}_{a}}{RT})$$

where *A* denotes the pre-exponential factor corresponding to the proton conductivity at infinite temperature, *σ*_T_ represents the ionic conductivity at absolute temperature, *E*_a_ is the activation energy, and *R* is the universal gas constant. The ionomer leaching behavior was evaluated under compressive stress or controlled humidity. Membranes (1 × 1 cm^2^) were first sandwiched between two gas-diffusion layers (GDLs) and pressed at 1 MPa and 30 °C for 5 min to ensure intimate contact. In a separate set of experiments, membrane pieces of the same size were exposed for 12 h to environments with relative humidities of 0, 11, 33, and 85%. For both tests, the leached ionomer content (wt%) was quantified from the mass change in the sample, by comparing the weight before (*W*_before_) and after (*W*_after_) the treatment according to the following expression.

(3) $$Leaching\;amount\left(wt\%\right)=\frac{\left(W_{after}-W_{before}\right)}{W_{before}}\times 100$$

Storage modulus of the samples (10 × 5.3 × 0.10 mm^3^) were tested by a dynamic mechanical analyzer (DMA, Q800, Waters, USA). The heating rate was set at 5 °C min^−1^ and frequency at 1 Hz.

## 3. Results and Discussion

### 3.1. Fabrication of Composite Membranes

**Figure 1:**
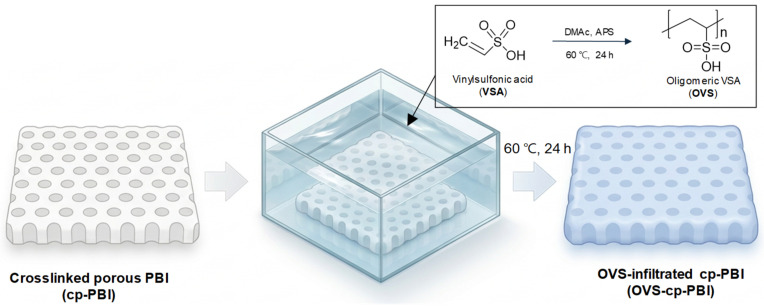
Schematic of the fabrication of OVS-cp-PBI membranes.

For OVS-cp-PBI, cp-PBI films were first prepared using our previously reported procedure [31]. The cp-PBI film was obtained by adding ZIF-8 into the PBI solution, followed by MDI as a crosslinker. The isocyanate groups of MDI reacted with the benzimidazole units of PBI and the imidazole groups of ZIF-8, yielding a covalently crosslinked network. During subsequent PA doping, ZIF-8, which is unstable in concentrated PA, underwent structural degradation and was removed, leaving behind a porous structure at the original ZIF-8 sites. Because a fraction of the imidazole groups remained covalently attached to the PBI backbone, the cp-PBI film exhibited crosslinked structure and increased basicity relative to pristine PBI. Owing to its basic, porous morphology and mechanically robust crosslinked framework, cp-PBI was employed as a host matrix for OVS infiltration (Figure 1). As a non-porous control, a dense PBI film, representative of typical HT-PEMs, was also fabricated (~50 μm of thickness).

### 3.2. Physicochemical Properties of OVS

**Figure 2:**
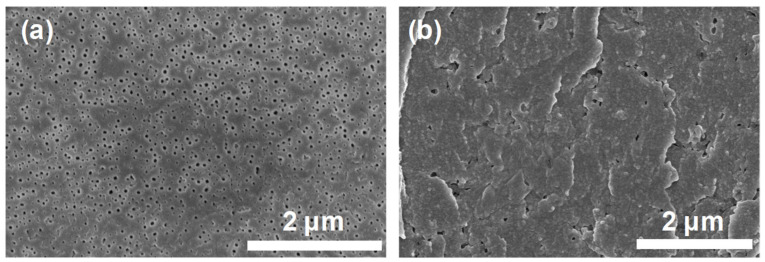
SEM images of (**a**) cp-PBI and (**b**) OVS-cp-PBI.

Polymerization of VSA was carried out in DMAc at 60 °C for 24 h in the presence of either cp-PBI or dense PBI (Figure 1). This process produced pore-filling membranes in which the pores of cp-PBI were infiltrated with OVS. SEM images reveal that the pores originally present in cp-PBI were completely filled with OVS, resulting in the formation of reinforced composite membranes (i.e., OVS-cp-PBI) (Figure 2).

In contrast, the dense PBI film exhibits only a thin coating layer of OVS on the surface (Figure S1). Consequently, the OVS uptake in cp-PBI reached approximately 71 wt%, which is about 1.5–fold larger than 48 wt% of the dense PBI membrane. Based on cross-sectional elemental analysis using EDS, sulfur signals originating from OVS were observed throughout the interior of the OVS-cp-PBI membrane, indicating uniform incorporation of OVS across the electrolyte matrix (Figure S2a). In contrast, only a limited amount of sulfur was detected within the OVS-infiltrated PBI membrane, suggesting that OVS penetration into the interior of dense PBI was restricted compared to cp-PBI, where OVS was effectively incorporated into the bulk of the membrane (Figure S2b).

**Figure 3:**
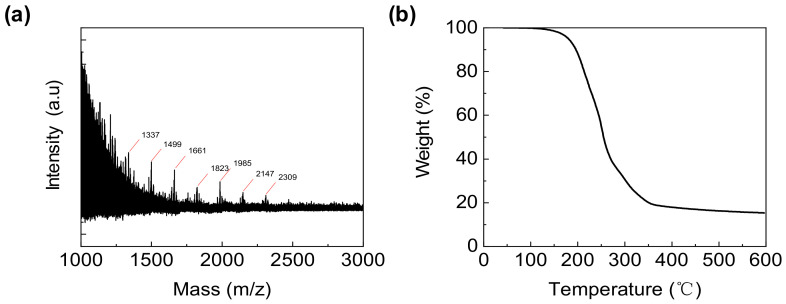
(**a**) MALDI-TOF MS of OVS. (**b**) TGA curve of OVS under N_2_ atmosphere.

APS is a highly efficient radical initiator in polar protic media such as water; however, in non-aqueous aprotic solvents like DMAc, the generated radicals can be stabilized or partially deactivated, reducing the effective initiation efficiency factor to about 0.3–0.5. This relatively low radical efficiency in DMAc suppresses excessive chain propagation during VSA polymerization, thereby favoring the formation of oligomeric VSA rather than high-molecular-weight poly(vinyl sulfonic acid). Taking this behavior into account, our goal was to obtain OVS as a wax-like oligomer that retains liquid-like proton transport while still possessing sufficient viscosity and dimensional integrity to form a solid film within the cp-PBI framework (Figure S3). MALDI-TOF MS analysis confirmed that the synthesized OVS consists of oligomers containing, on average, 10–19 VSA repeat monomeric units (Figure 3a).

The resulting composite electrolyte membranes can be used as intermediate-temperature (80–120 °C) PEMs without any additional proton-conducting medium, such as water or PA. Figure 3b shows the TGA curve of OVS recorded under nitrogen from room temperature to 600 °C at a heating rate of 10 °C min^−1^. Notably, OVS exhibits no significant thermal degradation up to approximately 180 °C. Owing to its fully aromatic backbone, the PBI host framework remains essentially stable with negligible mass loss until nearly 500 °C [32]. These results indicate that the combination of OVS and cp-PBI affords an OVS-cp-PBI with sufficient thermal robustness for use as a proton-conducting electrolyte in the intermediate-temperature range.

### 3.3. Proton Conductivity

**Figure 4:**
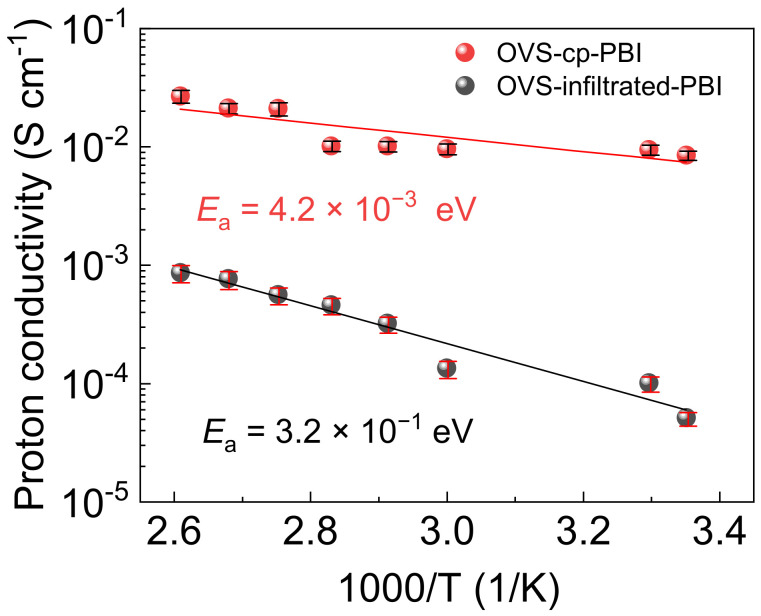
Average proton conductivity and activation energy of OVS-cp-PBI and OVS-infiltrated PBI.

VSA is a strong sulfonic acid with a pK_a_ of approximately 1.8–2.4, making it significantly stronger than acetic acid (pK_a_ ≈ 4.7) and formic acid (pK_a_ ≈ 3.7), but weaker than typical mineral acids such as sulfuric acid (pK_a_ ≈ −3.0) and hydrochloric acid (pK_a_ ≈ −6.3) [33]. This acidity implies that VSA readily dissociates to release protons in aqueous media, conferring pronounced acidic character to its oligomeric form, OVS. Despite being an oligomer, OVS therefore provides a high density of acid sites for proton generation and transport. The bulk resistance $R_{b}$ at each temperature was extracted from electrochemical impedance spectroscopy (EIS) using a simple equivalent circuit composed of $R_{b}$ in series with a constant phase element (CPE), and representative Nyquist plots together with the corresponding fits are provided in Figure S4. Proton conductivities were then calculated from the fitted $R_{b}$ values, and Figure 4 reports the average conductivity obtained from at least three independently prepared membranes, with error bars representing the standard deviation. As a result, the OVS-cp-PBI membrane exhibits high proton conductivity under both ambient and anhydrous conditions, showing an average values of 8.4 × 10^−3^ S cm^−1^ at room temperature without external humidification and 2.6 × 10^−2^ S cm^−1^ even at 110 °C under intermediate-temperature, water-free operation, which are comparable to or higher than typical PA-doped PBI membranes operated in the same temperature window but without the severe acid loss commonly observed in those systems (Figure 4). From the Arrhenius-type temperature dependence of the conductivity, the apparent activation energy for proton transport in OVS-cp-PBI was determined to be 4.2 × 10^−3^ eV, whereas that of the OVS-infiltrated PBI membrane was 3.2 × 10^−1^ eV (Figure 4). The extremely low activation energy of OVS-cp-PBI indicates an almost temperature-independent proton conduction pathway established by the confined oligomeric sulfonic acid phase, in sharp contrast to the much more thermally activated transport in OVS-infiltrated PBI.

These properties arise from the combination of the strong acidity of OVS and the substantial OVS uptake within the porous cp-PBI framework, which together create a percolated network of proton-conducting domains. In addition, the imidazole groups remaining in the cp-PBI matrix can participate in proton transport via a hopping (Grotthuss-type) mechanism, further contributing to the good anhydrous proton conductivity of OVS-cp-PBI in the intermediate-temperature range. In contrast, the OVS-infiltrated PBI membranes exhibited low proton conductivity of 5.0 × 10^−5^ and 8.5 × 10^−4^ S cm^−1^ at room temperature and 110 °C under anhydrous conditions, respectively (Figure 4). This is attributed to the limited distribution of the ionomer (OVS), which is primarily localized on the membrane surface rather than being uniformly incorporated throughout the bulk PBI matrix.

### 3.4. Ionomer Retention Behavior

**Figure 5:**
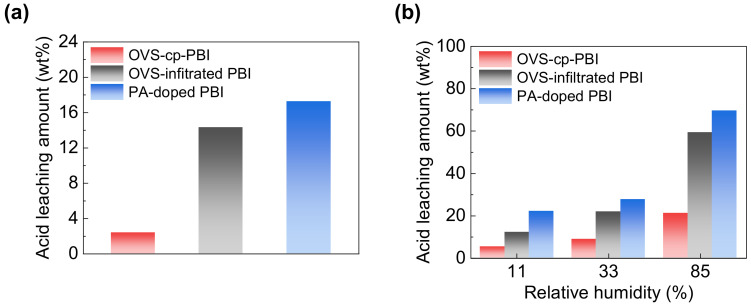
Ionomer leaching behavior of OVS-cp-PBI, OVS-infiltrated PBI, and PA-doped PBI membranes under (**a**) compressive stress or (**b**) controlled humidity.

Typical PA-doped PBI membranes can also achieve proton conductivities on the order of a few × 10^−3^ S cm^−1^ at room temperature under anhydrous conditions when a high PA content is incorporated [34]. However, due to the low molecular weight and strong hygroscopic nature of PA, these membranes suffer from significant acid leaching under such conditions, which remains a critical limitation. In addition to the strong acid–base interaction that promotes high OVS uptake in cp-PBI, the wax-like physical nature and high viscosity of OVS hinder its escape from the porous PBI framework once it is infiltrated. In conventional PA-doped PBI membranes, low-molecular-weight PA can readily leach out during membrane–electrode assembly (MEA) fabrication, owing to mechanical compression and the presence of water generated or supplied during fuel cell operation, which ultimately leads to performance decay. To compare the ionomer-retention capability of the two systems, OVS-cp-PBI, OVS-infiltrated PBI, and PA-doped PBI (PA uptake ~78.0 wt%) membranes were subjected to leaching tests under compressive stress or humidity-controlled conditions (Figure 5).

For the compression test, each membrane was placed between two GDLs and pressed at 1 MPa and 30 °C for 5 min, mimicking typical MEA hot-pressing conditions, and the amount of ionomer released from the membrane into the GDLs was quantified (Figure 5a). The average ionomer loss from the OVS-cp-PBI membrane was only 2.3 wt%, which is markedly lower than the 14 wt% and 17 wt% loss observed for the OVS-infiltrated PBI and PA-doped PBI membranes, respectively, indicating much stronger ionomer retention in the OVS-cp-PBI system.

Because acidic species interact strongly with water, PA leaching from PA-doped PBI under humid operating conditions is a well-recognized issue [35]. Therefore, we further evaluated ionomer stability under controlled relative humidity (RH) (Figure 5b). OVS-cp-PBI, OVS-infiltrated PBI and PA-doped PBI membranes (1 × 1 cm^2^ in size) were exposed for 12 h at RH = 0, 11, 33, and 85%, and the ionomer (i.e., OVS or PA) loss was determined from the mass change before and after exposure. Consistent with previous reports, the PA-doped PBI membrane exhibited severe PA leaching; for instance, at 85% RH, approximately 70 wt% of the acid ionomer was lost [36]. The OVS-infiltrated PBI membrane also showed considerable OVS loss of approximately 59 wt% at 85% RH. In contrast, the OVS-cp-PBI membrane showed only about 20 wt% OVS loss at the given condition, although a small amount of leaching still occurred. These results highlight that neither the porous morphology nor the acid functionality alone is sufficient to ensure durable IT-PEM operation. The synergy between the crosslinked porous PBI framework (providing strong basic sites and mechanical confinement) and the viscous oligomeric OVS phase (providing dense, yet less mobile, sulfonic acid sites) is crucial for simultaneously achieving high anhydrous conductivity and suppressed ionomer leaching.

### 3.5. Mechanical Properties

To evaluate the temperature-dependent mechanical stability of the OVS-cp-PBI membrane, DMA was conducted over a temperature range of –60~140 °C by monitoring the storage modulus (Figure S5). Although the storage modulus gradually decreased with increasing temperature, the OVS-cp-PBI membrane maintained a modulus on the order of a few of MPa even in the intermediate-temperature range of 80–120 °C. This result indicates that the membrane can retain sufficient mechanical integrity under elevated-temperature conditions, supporting its potential applicability as an electrolyte membrane for IT-PEMFCs.

## 4. Conclusions

In summary, an OVS-infiltrated crosslinked porous PBI membrane was successfully prepared as a high-performance electrolyte for IT-PEM applications. The ZIF-8-templated porous cp-PBI framework enabled high OVS uptake and uniform distribution within the membrane, forming an interconnected proton-conducting network. The oligomerized OVS, characterized by its wax-like and highly viscous nature, provided a high density of acid sites while effectively suppressing ionomer leaching. As a result, the OVS-cp-PBI membrane exhibited excellent proton conductivity under anhydrous conditions across a wide temperature range, including the intermediate-temperature regime (80–120 °C). Furthermore, the membrane demonstrated superior ionomer retention compared to conventional PA-doped PBI systems under mechanical compression or humid conditions, addressing one of the critical limitations of existing HT-PEMs. The enhanced stability originates from the strong acid–base interactions, the crosslinked structure of cp-PBI, and the reduced mobility of the oligomeric ionomer. Overall, this work provides a viable strategy for overcoming the trade-off between conductivity and durability in PEMs, offering a pathway toward stable, efficient, and humidity-independent electrolytes for next-generation fuel cell technologies.

## Data Availability

The raw data supporting the conclusions of this article will be made available by the authors on request.

## Supplementary Materials

The following supporting information can be downloaded at: https://www.mdpi.com/article/10.3390/polym18111298/s1, Figure S1: Cross-sectional SEM images of (a) PBI and (b) OVS-infiltrated PBI membranes; Figure S2: EDS spectra of (a) OVS-cp-PBI and (b) OVS-infiltrated PBI membranes; Figure S3: Photograph of wax-type OVS; Figure S4: Nyquist plots of OVS-cp-PBI at (a) RT and (b) 110 °C; Figure S5: Temperature-dependent storage modulus of the OVS-cp-PBI membrane.
