# Supplementary material for: Confinement of Oligomeric Vinyl Sulfonic Acid Within Crosslinked Porous Polybenzimidazole for Intermediate-Temperature Proton Exchange Membranes
Source: Polymers (Basel). 2026 May 25;18(11):1298. doi: 10.3390/polym18111298 (PMC13259151; doi:10.3390/polym18111298)
Supplement: Supplementary file 1 [file polymers-18-01298-s001.zip › polymers-4300739-supplementary.pdf]

Article

Confinement of Oligomeric Vinyl Sulfonic Acid Within Crosslinked Porous Polybenzimidazole for Intermediate-Temperature Proton Exchange Membranes

Hongbin Na and Sung-Kon Kim *

Department of Chemical and Biochemical Engineering, Dongguk University, Seoul 04620, Republic of Korea; 2026121074@dgu.ac.kr

* Correspondence: sungkonkim@dgu.ac.kr

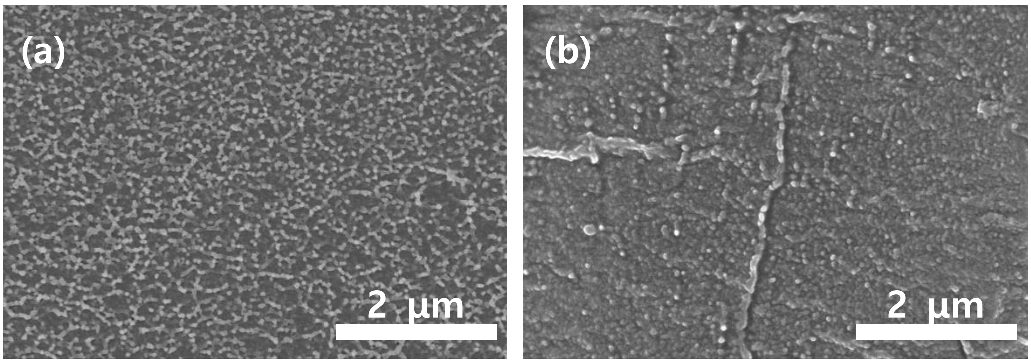

Figure S1. Cross-sectional SEM images of (a) PBI and (b) OVS-infiltrated PBI membranes.

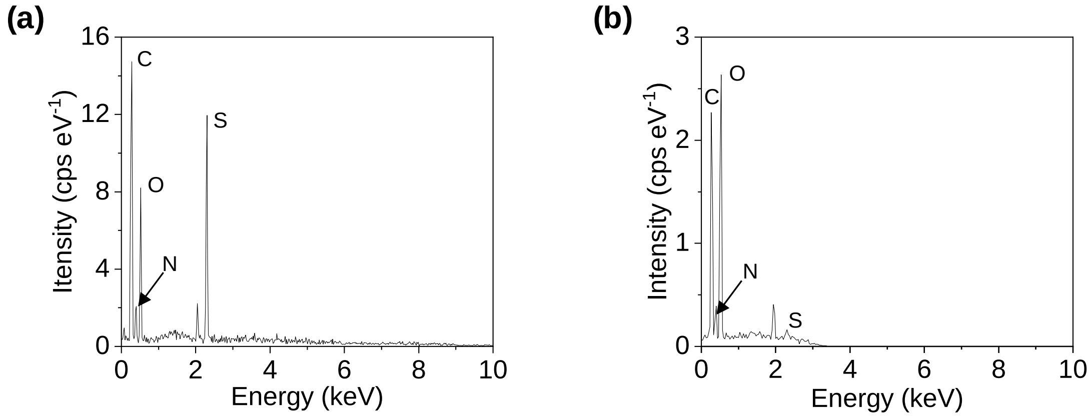

Figure S2. EDS spectra of (a) OVS-cp-PBI and (b) OVS-infiltrated PBI membranes.

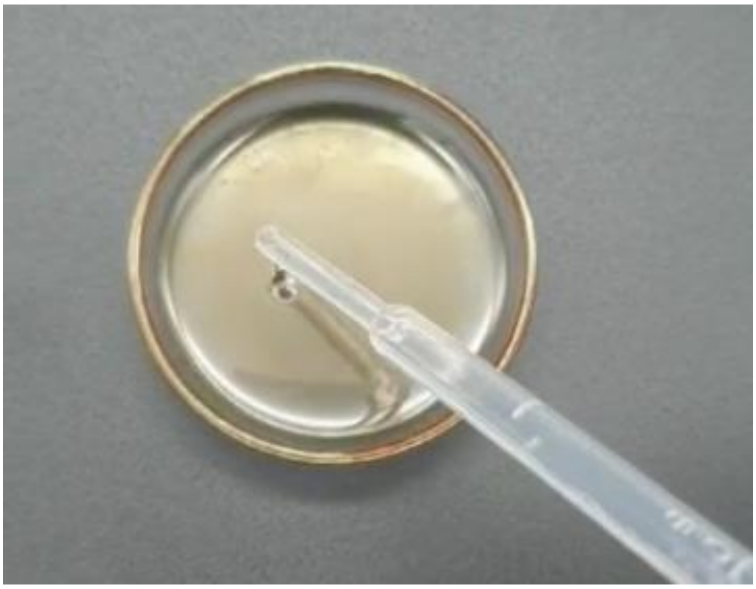

Figure S3. Photograph of wax-type OVS.

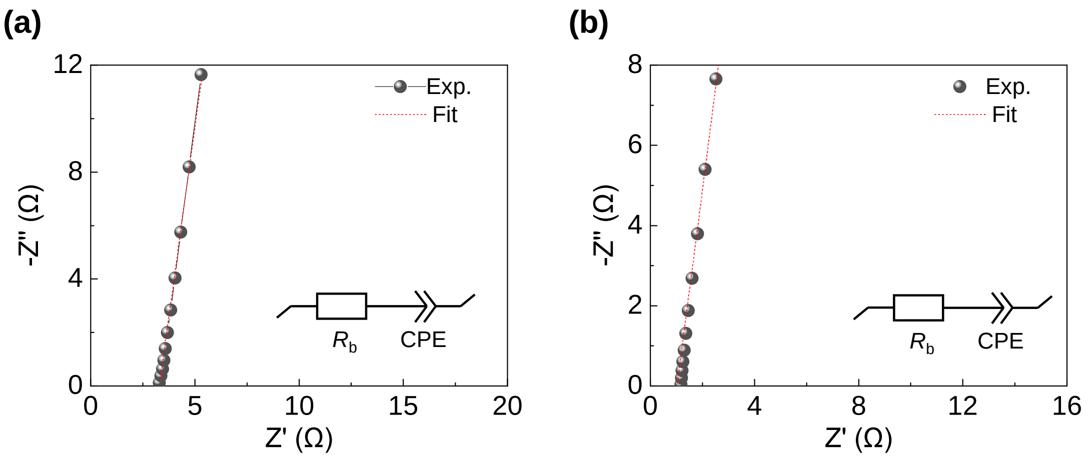

Figure S4. Nyquist plots of OVS-cp-PBI at (a) RT and (b) 110 °C.

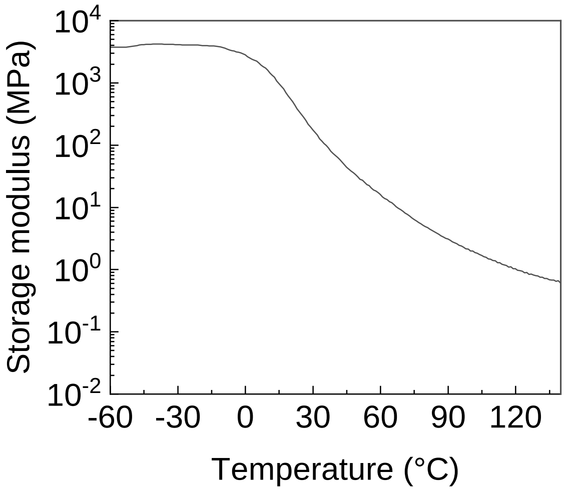

Figure S5. Temperature-dependent storage modulus of the OVS-cp-PBI membrane.
